# SINR-Based User Clustering for Downlink NOMA Systems with Limited Channel Information

**DOI:** 10.3390/s26103109

**Published:** 2026-05-14

**Authors:** Wonkyu Kim, Ngoc-Thanh Nguyen, Taehyun Jeon

**Affiliations:** 1 Department of Electrical and Information Engineering, Seoul National University of Science and Technology, Seoul 01811, Republic of Korea; 24520035@seoultech.ac.kr; 2 Faculty of Electrical and Electronic Engineering, PHENIKAA School of Engineering, PHENIKAA University, Hanoi 100000, Vietnam; thanh.nguyenngoc@phenikaa-uni.edu.vn

**Keywords:** non-orthogonal multiple access, mmWave, hybrid beamforming, clustering, power allocation

## Abstract

In next-generation wireless communication systems, spectrum efficiency can be realized through the integration of hybrid beamforming (HBF) and non-orthogonal multiple access (NOMA). To maximize the synergy between these two technologies, it is essential to accurately cluster users within beams. Most existing studies on clustering overlook practical constraints and assume perfect channel state information (CSI). However, obtaining full CSI is impractical in realistic environments due to high feedback overhead and potential CSI errors. To address these challenges, this paper adopts an opportunistic beamforming (OBF) framework based on a partial CSI environment. The OBF facilitates channel estimation and HBF precoder design using only signal-to-interference-plus-noise ratio (SINR) feedback. Subsequently, clustering and power allocation (PA) are performed utilizing the feedback SINR from OBF without requiring additional feedback information. While conventional NOMA focuses on maximizing either throughput or fairness, this paper proposes a scheme that selects users with high SINR to maximize system throughput while minimizing the throughput disparity among users to enhance fairness. Furthermore, a power allocation method that satisfies the minimum successive interference cancellation (SIC) power requirement is employed to ensure stable decoding. Simulation results demonstrate that the proposed clustering scheme enhances the sum-rate compared to conventional SINR-based clustering methods while maintaining fairness. Consequently, this study suggests a promising approach to improving NOMA performance in practical partial CSI environments.

## 1. Introduction

Non-orthogonal multiple access (NOMA) has emerged as a promising paradigm for next-generation wireless communication systems, offering enhanced spectral efficiency and the capacity to support massive connectivity. Unlike conventional orthogonal multiple access (OMA) schemes, which allocate orthogonal time or frequency resources to individual users, NOMA enables multiple users to simultaneously access the same resource block through power-domain multiplexing. At the receiver, successive interference cancellation (SIC) technique is employed to decode the superimposed signals, facilitating efficient multi-user detection and improved system throughput [1,2,3].

To achieve the performance gains of NOMA in multi-antenna systems, extensive research has been conducted on user clustering and power allocation (PA) strategies. User clustering determines which users are grouped within the same transmission resource, while PA assigns appropriate transmission power to each user within a cluster. Early studies demonstrated that pairing users with significantly different channel conditions can enhance SIC performance by allocating higher power to weaker users and lower power to stronger users [4,5,6]. To further optimize system performance in multi-user and multi-antenna environments, various clustering and resource allocation schemes have been proposed. For instance, joint approaches combining user clustering, beamforming, and PA have been investigated to maximize system throughput in downlink multi-user multiple-input multiple-output (MU-MIMO) NOMA systems [5]. Additionally, resource allocation strategies considering proportional rate constraints in hybrid multiple access systems have been explored [6].

With the increasing demand for high data rates and expanded bandwidth, millimeter-wave (mmWave) communication has emerged as a key architecture for next-generation wireless networks. However, due to severe path loss and the directional propagation characteristics of the mmWave band, advanced beamforming techniques are essential to maintain stable communication links [1]. Hybrid beamforming (HBF), which combines analog and digital beamforming architectures, is widely applied in mmWave systems to achieve high gains while reducing hardware complexity. Beyond communication performance, the fundamental limitations of mmWave systems have also been studied in terms of localization. For example, sub-meter positioning accuracy can be achieved in linear topologies even with a limited anchor nodes, owing to the high directionality of mmWave antenna arrays [7]. These foundational studies emphasize the multi-faceted importance of beamforming and antenna array configurations. Furthermore, opportunistic beamforming (OBF) techniques are attractive for practical multi-user systems as they effectively exploit multi-user diversity with limited channel feedback [8,9].

Various studies have investigated the integration of NOMA and HBF in mmWave systems, often assuming the availability of accurate channel state information (CSI). For instance, a joint optimization framework was proposed to enhance energy efficiency in multi-user mmWave NOMA systems by employing channel correlation and the k-means algorithm for user grouping, alongside simultaneous beamforming and PA [10]. Similarly, an opportunistic beam-splitting NOMA scheme was analyzed for mmWave networks, leveraging distance-based clustering and beam-domain multiplexing under the assumption of known base station (BS)-to-user distance [11]. In [12], a joint resource allocation strategy for multi-user OBF using OFDM-NOMA systems was investigated, exploring all possible user combinations to optimize spectral efficiency (SE). Furthermore, clustering methods have been actively applied across various domains, including k-means-based secure clustering for IoT environment [13] and adaptive clustering for UAV-assisted NOMA systems [14].

Other research has extended traditional channel-based clustering [4,5,6] to NOMA-based mobile edge computing (MEC) networks to develop energy-efficient (EE) resource allocation approach [15]. Additionally, the use of channel correlation-as seen in [4,10]-within the agglomerative nesting (AGNES) algorithm has been shown to imporve performance [16]. Despite these advancements, most existing studies rely on the assumption of perfect CSI at the transmitter. In practical wireless systems, however, acquiring full CSI is challenging due to feedback overhead, hardware constraints, and channel estimation errors.

Consequently, research on user clustering and resource allocation under imperfect or partial CSI conditions has been recently gained momentum. For example, a two-stage hierarchical clustering study utilized a coalitional formation game algorithm and non-coherent joint transmission to improve transmission efficiency [17]. Moreover, a cross-entropy-based optimization has been investigated for NOMA networks with uncertain channel information [18]. Furthermore, a limited feedback-based clustering scheme utilizing partial CSI parameters, such as channel gain and Angle of Departure (AoD), has been proposed specifically for mmWave NOMA systems [19].

Among various approaches, OBF has emerged as an effective strategy for mmWave systems operating under partial CSI, as it primarily relies on signal-to-interference-plus-noise ratio (SINR) feedback rather than explicit CSI. In OBF-based systems, SINR feedback is essential for supporting proportional fair (PF) scheduling, which ensures long-term fairness in user selection. Therefore, it is desirable to design clustering strategies based solely on SINR feedback to maintain compatibility with the OBF framework. While conventional NOMA systems often employ “high-low” user pairing to ensure SIC stability, this can lead to severe throughput imbalances within a cluster. To overcome this, a strategy that enhances system throughput while maintaining robust user fairness is required.

This paper proposes a balanced clustering and PA framework for mmWave NOMA systems utilizing partial CSI. Specifically, the proposed approach adopts a clustering strategy that groups users with favorable channel conditions and applies a dedicated PA method to ensure reliable SIC decoding. Distinguishing itself from conventional pairing, this approach improves intra-cluster fairness, as measured by Jain’s fairness index, while maintaining competitive system throughput. Furthermore, the proposed framework is integrated with an adaptive perturbation-aided opportunistic hybrid beamforming (AP-OHBF) scheme, where the precoder is updated based only on SINR feedback. This design enables an OBF-NOMA system that implements feedback-based scheduling and clustering without additional overhead. While [7] presents theoretical limits of localization, this study focuses on the communication perspective. Consequently, the proposed approach achieves a balanced performance in terms of throughput and fairness while preserving the inherent characteristics of PF scheduling.

The remainder of this paper is organized as follows. Section 2 describes the mmWave NOMA system model and formulates the balanced NOMA clustering and PA problem under limited channel information. Section 3 presents the proposed balanced clustering algorithm along with the hybrid beamforming and PA schemes. Section 4 provides simulation results to evaluate the performance of the proposed method in realistic mmWave channel environments. Finally, Section 5 concludes the paper. The key notations used in this study are listed in Table A1 for ease of reference.

## 2. System Model

**Figure 1:**
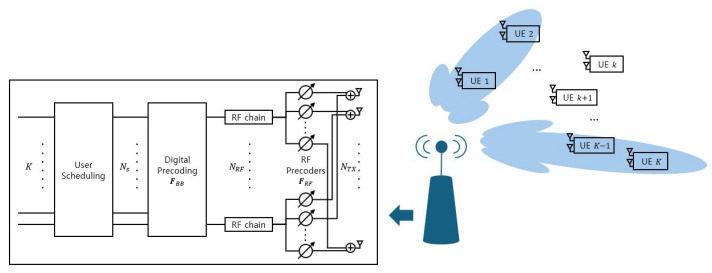
Hybrid Beamforming-Based Downlink MU-MIMO-NOMA system Model.

Figure 1 illustrates a downlink MU-MIMO-NOMA system operating over a mmWave channel. The base station (BS) adopts a fully connected HBF architecture with $N_{T}$ transmit antennas and $N_{\mathrm{RF}}$ radio frequency (RF) chains. Each RF chain is connected to all transmit antennas through a network of phase shifters. Each user is equipped with $N_{R}$ receive antennas connected to a single RF chain.

Each user belongs to only one cluster, and each cluster is served by a single beam. Since a beam can be formed by one or more RF chains, the number of clusters satisfies $C\leq N_{\mathrm{RF}}$. To serve the *C* clusters, the BS transmits $N_{s}$ ($N_{s}=C$) independent data streams through the available RF chains, ensuring that the number of transmitted streams does not exceed $N_{\mathrm{RF}}$. A total of *K* users are partitioned into *C* clusters, and each cluster contains at least two users to facilitate NOMA transmission. Let $K_{n}$ denote the number of users in the *n*-th cluster.

The SINR of the *k*-th user in the *n*-th cluster is given by

(1) $$SINR_{n,k}=\frac{P_{n,k}{\left|w_{n,k}^{H}H_{n,k}f_{n}\right|}^{2}}{\sum_{i=1}^{k-1}P_{n,i}{\left|w_{n,k}^{H}H_{n,k}f_{n}\right|}^{2}+\sum_{\begin{array}{c} j=1\\ j\neq n \end{array}}^{C}\sum_{i=1}^{K_{j}}P_{j,i}{\left|w_{n,k}^{H}H_{n,k}f_{j}\right|}^{2}+\sigma^{2}},$$

where $w_{n,k}$, $H_{n,k}$, and $P_{n,k}$ denote the receive combining vector, channel matrix, and allocated transmit power of the *k*-th user in the *n*-th cluster, respectively. The vector $f_{n}$ represents the precoding vector associated with the *n*-th cluster, and $\sigma^{2}$ denotes the noise variance. In the denominator, the first term represents intra-cluster interference, the second term accounts for inter-cluster interference, and the final term corresponds to additive white gaussian noise (AWGN). The primary performance metric of a NOMA system is the overall system throughput, defined as

(2) $$R_{\mathrm{sum}}=\sum_{n=1}^{C}\sum_{k=1}^{K_{n}}B\log_{2}\left(1+SINR_{n,k}\right),$$

where *B* denotes the system bandwidth and $SINR_{n,k}$ is the achievable SINR of the *k*-th user in the *n*-th cluster. This paper considered two measures: self-fairness and Jain’s fairness index. When the total number of users *K* exceeds the simultaneous serving capacity of the BS, scheduling becomes necessary, potentially leading to unfairness. The self-fairness metric is defined as [8,19]:

(3) $$F_{\mathrm{self}}=-\frac{1}{\log K}\sum_{k=1}^{K}p_{k}\log p_{k},$$

where $p_{k}$ is the probability that the *k*-th user is selected for transmission. This entropy-based metric approaches unity when users are selected with equal probability. Furthermore, to quantify the throughput disparity within a cluster caused by PA and channel conditions, Jain’s fairness index is adopted [20]:

(4) $$J=\frac{{\left(\sum_{n=1}^{C}\sum_{k=1}^{K_{n}}R_{n,k}\right)}^{2}}{C\sum_{n=1}^{C}\sum_{k=1}^{K_{n}}R_{n,k}^{2}}.$$

This index approaches unity as users within a cluster achieve similar throughput and decrease toward zero as the disparity increases. In practical systems, the BS typically estimates user SINR based on effective channel gain (EchG) feedback. Motivated by this, the proposed model assume an SINR-feedback-based framework, where clustering and transmission decisions rely solely on user-reported SINR values rather than full CSI.

**Figure 2:**
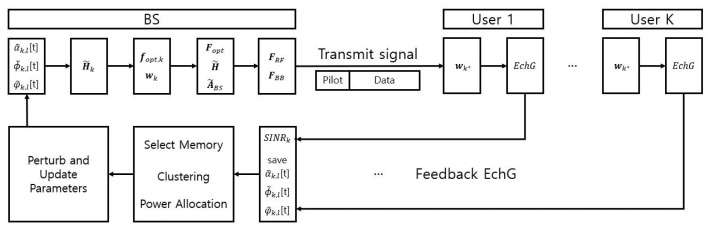
Sequential procedure of channel tracking, hybrid beamforming, and memory-based user clustering in the proposed system.

The AP-OHBF scheme, as proposed for partial CSI environments in [8,19], designs precoders by estimating channel parameters and constructing an effective channel model based on these estimates. Initiallly, the channel parameters are randomly initialized. At each time slot, a perturbation vector with random direction and fixed magnitude is added to update these parameters. A beamforming precoder is then generated using the updated parameters, and pilot signals are transmitted. The system observes the SINR feedback from users. If the updated parameters yield improved SINR performance, the perturbed parameters are accepted; otherwise, the previous values are retained. As illustrated in Figure 2, this iterative process facilitates adaptive beamformer refinement under partial CSI conditions using only SINR feedback.

When users with consistently high SINR values are repeatedly selected, system resources may be concentrated on a limited subset, leading to unfairness. To mitigate this, a PF-metric is adopted as in [8], defined as:

(5) $${\mathrm{PF}}_{k,s}^{\left(m\right)}=\frac{R_{k,s}^{\left(m\right)}}{{\bar{R}}_{k}},$$

where $R_{k,s}^{\left(m\right)}$ denotes the throughput of the *k*-th user on the *s*-th data stream stored in the *m*-th memory slot. Here, $N_{s}=\min\left(K,N_{\mathrm{RF}}\right)$ represents the total number of data streams, and *M* denotes the memory size for storing recent throughput values. The average throughput of the *k*-th user, ${\bar{R}}_{k}$, is updated at each time slot as:

(6) $${\bar{R}}_{k}\left[t+1\right]=\left\{\begin{array}{cc} \left(1-\frac{1}{T_{c}}\right){\bar{R}}_{k}\left[t\right]+\frac{1}{T_{c}}R_{k}\left[t\right], & \;\mathrm{if}\;k\in C\\ \left(1-\frac{1}{T_{c}}\right){\bar{R}}_{k}\left[t\right], & \;\mathrm{if}\;otherwise \end{array}\right.$$

where $T_{c}$ is a time constant controlling the trade-off between multi-user diversity gain and clustering latency, and C denotes the set of users selected in the current time slot. As the throughput of selected users accumulates in ${\bar{R}}_{k}$, their PF-metric values decrease, thereby preventing repeated scheduled of the same users and improving long-term fairness.

Specifically, clustering is performed by selecting the top *M* SINR values for each RF chain based on the SINR feedback. To ensure that each user belongs to exatly one cluster, a user assigned to a specific RF chain (cluster) is excluded from other clusters. If all possible user combinations were exhaustively searched to determine the optimal clustering configuration, a total of combinations would need to be evaluated, leading to prohibitively high computational complexity. To address this, this paper proposes a memory-based greedy clustering algorithm that significantly reduces computational complexity while maintaining effective performance.

## 3. SINR-Based Joint Clustering and Power Allocation

### 3.1. Throughput and Fairness Balanced User Clustering

Algorithm 1 summarizes the proposed memory-based greedy clustering procedure. In this algorithm, Γ denotes the sum-PF-metric, while U, S, and C represent the sets of users, data streams (RF chains), and clusters, respectively. In step 7, the PF-metric for each user is calculated based on the received feedback. Subsequently, a greedy clustering process is executed from step 8 to 21. Specifically, step 10–13 identify the user with the maximum SINR for a given RF chain to serve as the cluster head. Step 14–18 then select a second user from the updated set U to complete the cluster. This iterative mechanism prevents the redundant selection of the same user and ensures that users with superior channel conditions are assigned to each RF chain.
**Algorithm 1** Throughput and Fairness Balanced User Clustering Algorithm  1:**Input:** ${\mathrm{SINR}}_{1},\ldots ,{\mathrm{SINR}}_{M}\in R^{K\times N_{s}}$  2:**Output:** $C_{i}\in R^{1\times K_{i}}$$\left(i=1,\ldots ,N_{s}\right)$, m*  3:Initialize $\Gamma_{\max}=0$  4:**for** m=1 to *M* **do**  5: Initialize Γ=0  6: U={1,…,K}, $S=\{1,\ldots ,N_{s}\}$  7: ${\mathrm{PF}}_{k,i}^{\left(m\right)}=\frac{{\mathrm{SINR}}_{k,i}^{\left(m\right)}}{T_{k}}$  8: **for** i=1 to $\min(N_{RF},K)$ **do**  9:  **for** j=1 to $M_{i}$ **do**10:   **if** j=1 **then**11:    (i*,k*$)=\arg\max_{k\in U,\;i\in S}{\mathrm{PF}}_{k,i}^{\left(m\right)}$12:    $C_{i^{*},j}\leftarrow k^{*}$13:    U←U∖k*14:   **else**15:    k** $=\arg\max_{k\in U}{\mathrm{PF}}_{k,i^{*}}^{\left(m\right)}$16:    $C_{i^{*},j}\leftarrow k^{**}$17:    U←U∖k**18:   **end if**19:  **end for**20:  S←S∖i*21: **end for**22: $\Gamma =\sum_{i=1}^{\min(N_{RF},K)}\sum_{j=1}^{K_{i}}{\mathrm{PF}}_{C_{i,j},i}^{\left(m\right)}$23: **if** $\Gamma_{\max}<\Gamma$ **then**24:  $\Gamma_{\max}\leftarrow \Gamma$25:  m* ←m26: **end if**27:**end for**

To evaluate the computational efficiency, we compare the proposed greedy approach with an exhaustive search clustering. Without optimization, exhaustive search requires examing all possible disjoint user combinations, resulting in a complexity of OM·∏n=0Nrf−1K−nCC≈O(M·KC·Nrf). Even when optimized using bitmask dynamic programming to avoid redundant state evaluations, the complexity remains $O(M\cdot N_{rf}\cdot 2^{K})$, which still grows exponentially with the number of users *K*. In contrast, the proposed greedy algorithm sequentially selects the best *C* users for each RF chain. This reduces the search space to a sum of combinations, OM·∑n=0Nrf−1K−nCC≈O(M·Nrf·KC). For a fixed cluster size *C*, the greedy approach follows polynomial-time complexity, offering a scalable alternative for large-scale systems by effectively balancing the trade-off between global optimality and computational overhead.

Step 22 computes the aggregate PF-metric, and step 23–26 identify the optimal configuration among the current and previous M−1 memory instances. This look-back approach aims to maximize system throughput by selecting the configuration with the most accurate channel estimation. Given a coherence time of $T_{c}=1000$ slots and the presence of user mobility, the memory size M=20 represents a sufficiently short interval to ensure that the stored channel parameters remain highly correlated with the current spatial environment. Rather than relying on stagnant historical data, this mechnism provides reliable starting points for the adaptive perturbation process, allowing the system to track the evolving optimal beams of mobile user effectively. Since the memory is updated only when a superior aggregate PF-metric is achieved, the greedy process inherently filters out outdated configurations and maintains effective parameters for high-density and mobile scenarios.

### 3.2. Power Allocation for SIC-Constrained NOMA

To ensure successful SIC decoding, appropriate PA must be performed after user clustering and prior to signal superposition. For the *n*-th cluster containing $K_{n}$ users, the throughput maximization problem is formulated as:

(7) $$\max_{p}\;\sum_{k=1}^{K_{n}}B\log_{2}(1+\frac{g_{n,k}p_{n,k}}{g_{n,k}\sum_{j=1}^{k-1}p_{n,j}+\sigma^{2}})$$

(8) $$s.t.\;C_{1}:\;\sum_{k=1}^{K_{n}}p_{n,k}\leq p_{n}$$

(9) $$C_{2}:\;B\log_{2}(1+\frac{g_{n,k}p_{n,k}}{g_{n,k}\sum_{j=1}^{k-1}p_{n,j}+\sigma^{2}})\geq R_{n,k},\;\forall k$$

(10) $$C_{3}:\;\left(p_{n,k}-\sum_{j=1}^{k-1}p_{n,j}\right)g_{n,k-1}\geq p_{\mathrm{tol}},\;\forall k\neq 1$$

where $P_{tol}$ denotes the minimum required difference in received power between users within the same cluster to facilitate robust SIC. The optimization is subject to the following fundamental constraints: $C_{1}$ regulates the total transmit power allocated to each cluster to stay within a predefined power budget; $C_{2}$ guarantees the quality of service (QoS) by satisfying the minimum required data rate for each individual user; $C_{3}$ enforces the power-domain constraints necessary to maintain a reliable SIC decoding order. The EchG is defined as:

(11) $$g_{n,k}={\left|w_{n,k}^{H}H_{n,k}f_{n}\right|}^{2},$$

where *B* is transmission bandwidth and $\sigma^{2}$ is noise power.

In the proposed system, the optimal PA for 1st user of n-th can be expressed:

(12) $$\begin{array}{cc} p_{n,1} & =\frac{p_{n}}{\prod_{\underset{j\notin B^{\prime }}{j=2}}^{K_{n}}\varphi_{n,j}\prod_{\underset{j\in B^{\prime }}{j=2}}^{K_{n}}2}-\sum_{\underset{j\notin B^{\prime }}{j=2}}^{K_{n}}\frac{\left(\varphi_{n,j}-1\right)}{g_{n,j}\prod_{\underset{j^{\prime }\notin B^{\prime }}{j^{\prime }=2}}^{j}\varphi_{n,j^{\prime }}\prod_{\underset{j^{\prime }\in B^{\prime }}{j^{\prime }=2}}^{j}2}\\ & -\sum_{\underset{j\notin C^{\prime }}{j=2}}^{K_{n}}\frac{P_{tol}}{2g_{n,j-1}\prod_{\underset{j^{\prime }\notin B^{\prime }}{j^{\prime }=2}}^{j-1}\varphi_{n,j^{\prime }}\prod_{\underset{j^{\prime }\in B^{\prime }}{j^{\prime }=2}}^{j-1}2}. \end{array}$$

where $B^{\prime }$ and $C^{\prime }$ represent the complementary sets associated with the minimum rate requirement and SIC robustness constraints, respectively, as formally defined [5]. Under these conditions, for $k\notin B^{\prime }$, the optimal PA for the k-th user within the n-th cluster is derived as:

(13) $$\begin{array}{cc} p_{n,k}= & [\frac{p_{n}}{\prod_{\underset{j\notin B^{\prime }}{j=k}}^{K_{n}}\varphi_{n,j}\prod_{\underset{j\in B^{\prime }}{j=k}}^{K_{n}}2}-\sum_{\underset{j\notin B^{\prime }}{j=k}}^{K_{n}}\frac{\left(\varphi_{n,j}-1\right)}{g_{n,j}\prod_{\underset{j^{\prime }\notin B^{\prime }}{j^{\prime }=k}}^{j}\varphi_{n,j^{\prime }}\prod_{\underset{j^{\prime }\in B^{\prime }}{j^{\prime }=k}}^{j}2}\\ & -\sum_{\underset{j\notin C^{\prime }}{j=k}}^{K_{n}}\frac{P_{tol}}{2g_{n,j-1}\prod_{\underset{j^{\prime }\notin B^{\prime }}{j^{\prime }=k}}^{j-1}\varphi_{n,j^{\prime }}\prod_{\underset{j^{\prime }\in B^{\prime }}{j^{\prime }=k}}^{j-1}2}+\frac{1}{g_{n,k}}]\times \left(\varphi_{n,k}-1\right). \end{array}$$

On the other hand, for the case where $k\in B^{\prime }$, the optimal PA is given by the following closed-form expression:

(14) $$\begin{array}{cc} p_{n,k}= & \frac{p_{n}}{\prod_{\underset{j\notin B^{\prime }}{j=k}}^{K_{n}}\varphi_{n,j}\prod_{\underset{j\in B^{\prime }}{j=k}}^{K_{n}}2}-\sum_{\underset{j\notin B^{\prime }}{j=k}}^{K_{n}}\frac{\left(\varphi_{n,j}-1\right)}{g_{n,j}\prod_{\underset{j^{\prime }\notin B^{\prime }}{j^{\prime }=k}}^{j}\varphi_{n,j^{\prime }}\prod_{\underset{j^{\prime }\in B^{\prime }}{j^{\prime }=k}}^{j}2}\\ & -\sum_{\underset{j\notin C^{\prime }}{j=k}}^{K_{n}}\frac{P_{tol}}{2g_{n,j-1}\prod_{\underset{j^{\prime }\notin B^{\prime }}{j^{\prime }=k}}^{j-1}\varphi_{n,j^{\prime }}\prod_{\underset{j^{\prime }\in B^{\prime }}{j^{\prime }=k}}^{j-1}2}+\frac{P_{tol}}{g_{n,k-1}}. \end{array}$$

This PA design ensures that both QoS requirements and SIC constraints are satisfied while maintaining low computational complexity serving as a critical component for reliable NOMA transmission under partial CSI.

### 3.3. AP-OHBF-Based NOMA System

To support efficient multi-user transmission under partial CSI, the proposed clustering and PA strategies are integrated into an AP-OHBF framework. The AP-OHBF scheme is particularly suitable for mmWave systems, where acquiring full instantaneous CSI is challenging due to high training overhead and hardware constraints. In the considered system, the BS relies on tracked channel parameters and limited feedback, such as EchGs and SINR values, instead of full CSI. Based on this information, analog and digital beamformers are jointly designed using hybrid precoding. Specifically, the RF precoder $F_{RF}$ is constructed from a predefined codebook, while the baseband precoder $F_{BB}$ is obtained using an MMSE-based approach to mitigate inter-cluster interference.

Unlike conventional AP-OHBF schemes designed for OMA systems, the proposed framework incorporates a clustering mechanism tailored for NOMA operation. In particular, users are grouped based on their effective channel conditions and compatibility for SIC decoding, replacing the user scheduling step in conventional AP-OHBF algorithm. Furthermore, the proposed PA strategy is applied within each cluster to satisfy SIC constraints and minimum rate requirements. The use of EchGs in both clustering and PA ensures consistent decision-making under partial CSI. The overall operation is summarized in Algorithm 2. Compared to the conventional AP-OHBF, the proposed scheme introduces two key modifications: (1) a NOMA-specific clustering-based grouping strategy and (2) a SIC-aware PA procedure. The detailed operational procedures of Algorithm 2 are elaborated as follows:

Step 1–3 (Initialization and Tracking): At the initial time slot (t=0), the BS initializes the tracked channel parameters $\left\{{\hat{\alpha }}_{k,l},{\hat{\phi }}_{k,l},{\hat{\varphi }}_{k,l}\right\}$, where path gains follow CN(0,1) and angles are uniformly distributed in a *Q*-bit quantized set [0,2π). The BS constructs the tracked channel matrix ${\hat{H}}_{i}$ and derives the optimal unconstrained precoder $f_{opt,i}$ via singular value decomposition (SVD). An analog combiner ${\hat{w}}_{i}$ is then selected from the RF codebook $W_{RF}$ to maximize the effective gain, i.e., ${\hat{w}}_{i}=\arg\max_{w_{i}\in W_{RF}}∥w_{i}^{H}{\hat{H}}_{i}∥$.

Step 4–5 (Hybrid Precoding and Hardware Constraints): Utilizing the hybrid MMSE precoding framework [21], the BS generates $F_{RF}$ and $F_{BB}$ at the time-slot level. To accommodate the hardware limitations of fully-connected HBF architectures, this update frequency aligns with the microsecond-range switching speeds of modern mmWave phase shifters [8]. Furthermore, the incremental perturbation strategy ensures only minor phase adjustments per slot, significantly reducing hardware settling time and power consumption compared to full re-computation.
**Algorithm 2** AP-OHBF-based NOMA system**Initialization:**  1:At t=0, the BS generates the initial tracked channel parameters:${\hat{\alpha }}_{k,l}\left[0\right]∼\mathrm{CN}\left(0,1\right),\left\{{\hat{\phi }}_{k,l}\left[0\right],{\hat{\varphi }}_{k,l}\left[0\right]\right\}∼U\left(0,2\pi \right)$.**Iterative Update**  2:The BS obtain tracked channel:${\hat{H}}_{i}=\sqrt{\frac{N_{T}N_{R}}{L}}\sum_{l=1}^{L}\alpha_{k,l}\left[t\right]a_{R}\left(\phi_{k,l}\left[t\right]\right)a_{T}\left(\varphi_{k,l}\left[t\right]\right)$.  3:The BS obtains the optimal unconstrained precoder and choose combiner:$f_{opt,i}=V_{i}^{\left(l\right)}$, $\hat{w_{i}}=arg\max_{w_{i}\in W_{\mathrm{RF}}}∥w_{i}^{H}{\hat{H}}_{i}∥$.  4:The BS generate $F_{RF}$ and $F_{BB}$ using hybrid MMSE precoding in [21].  5:The BS transmit pilot signals to all users applying $F_{RF}$ and $F_{BB}$.  6:Each user determine RF combiner $w_{k}$ to measure EchG (11) and then feed them back to the BS.  7:The BS calculate $SINR_{n,k}$ in (1) and store them with ${\hat{\alpha }}_{k,l}$, ${\hat{\phi }}_{k,l}$, ${\hat{\varphi }}_{k,l}$, $F_{RF}$ and $F_{BB}$ in the memory. $PF_{n,k}^{m}$ is calculated as (5).  8:By using Algorithm 2, the BS selects a set of clustered users and memory slot $m^{*}$.  9:Allocate transmit power using Table 1.10:The BS utilized $F_{RF}$, $F_{BB}$ and allocated power in the selected memory slot transmit data to the clustered users and updated $T_{k}$ according to (6).11:Update the tracked channel parameters, ${\hat{\alpha }}_{k,l}\left[t\right]$, ${\hat{\phi }}_{k,l}\left[t\right]$ and ${\hat{\varphi }}_{k,l}\left[t\right]$.12:Apply the perturbation on the tracked channel parameters to obtain channel parameters ${\hat{\alpha }}_{k,l}\left[t+1\right]$, ${\hat{\phi }}_{k,l}\left[t+1\right]$ and ${\hat{\varphi }}_{k,l}\left[t+1\right]$.13:Go to step 2 with t←t+1.

**Table 1:** System Summary of related works on user clustering strategies based on CSI types.

Ref.	CSI Type	Clustering Strategy	Key Contribution
[5]	Full CSI	Correlation, Channel Gain	Channel gain-based low-high clustering
[6]	Full CSI	Channel Gain	Power allocation (PA) under SIC constraints
[10]	Full CSI	Correlation, K-means	Joint optimization of HBF precoder and PA
[11]	Full CSI	Distance-based	Opportunistic beam-splitting NOMA
[12]	Full CSI	Heuristic (Max SE)	SE optimization in multi-carrier systems
[17]	Partial CSI	CFG, NC-JT	Enhancement of transmission efficiency
[18]	Partial CSI	Cross-Entropy	Maximum throughput optimization
[19]	Partial CSI	AoD, Channel Gain	Limited feedback-based clustering (1-bit)
**Proposed**	**Partial CSI**	**SINR-based Greedy Clustering**	**Balanced clustering without additional overhead**

Step 6–7 (Sequential Feedback and Centralized SINR Calculation): To address the lack of detailed multi-beam interference handling in conventional schemes, we define a sequential feedback mechanism. Each user reports its measured EchG, allowing the BS to centrally calculate the SINR by incorporating inter-cluster interference profiles from the preceding slot. This centralized approach ensures the accuracy of the PF-metric even in an iterative beam-update environment, effectively mitigating interference mismatch.

Step 8–10 (Memory-based Clustering and NOMA Integration): The BS identifies the optimal memory slot $m^{*}$ that maximizes the aggregate PF-metric. Given the coherence time and user mobility, the memory size *M* is selected to ensure that stored parameters remain highly correlated with the spatial environment. Since the memory is updated only upon achieving superior performance, this greedy approach filters out outdated data. Subsequently, NOMA-specific clustering and SIC-aware PA are executed based on $m^{*}$.

Step 11–13 (Adaptive Perturbation and Iterative Update): Random perturbations $\left\{\Delta \hat{\alpha },\Delta \hat{\phi },\Delta \hat{\varphi }\right\}$ are applied to the parameters of the selected slot. Specifically, the path gain and angle are updated as $\hat{\phi }\left[t+1\right]=\hat{\phi }\left[t\right]+\Delta \hat{\phi }$, $\hat{\varphi }\left[t+1\right]=\hat{\varphi }\left[t\right]+\Delta \hat{\varphi }$, and $\hat{\alpha }\left[t+1\right]=\rho \hat{\alpha }\left[t\right]+\sqrt{1-\rho^{2}}\Delta \hat{\alpha }$ where $\rho =J_{0}\left(2\pi f_{D}T\right)$ as in [22]. This adaptive strategy adopts only those perturbations that improve throughput, enabling the system to track optimal beams for mobile users while maintaining robustness through historical best-performing configurations in the memory.

## 4. Simulation and Results

In this section, we evaluate the performance of the proposed balanced NOMA clustering scheme through numerical simulations. The proposed shceme is compared with three reference frameworks:

- Geometry-aware Clustering [19]: In this partial CSI environment, users with similar AoD are grouped to form clusters.
- Channel-aware Clustering [5,6]: Users are grouped based on the EchG used in SINR calculation, typically pairing high-gain and low-gain users to enhance SIC performance.
- OMA [3]: A conventional time-division multiple access (TDMA) system is employed to verify the efficacy of intra-beam interference elimination via SIC in NOMA.

To ensure a fair comparison, all schemes are evaluated under identical feedback and computational constraints. Specifically, every NOMA clustering strategy—including the proposed, geometry-aware, and channel-aware schemes—operates within the same AP-OHBF framework and utilizes the same total power budget. Furthermore, all schemes are restricted to partial CSI feedback; while the benchmarks require specific parameters such as AoD or explicit channel gains, our proposed method demonstrates superior or competitive performance using only SINR feedback, which inherently implies a more efficient use of the available feedback overhead. The simulations assume a slow-fading environment where each user’s AoD and angle of arrival (AoA) vary within a predefined range. The primary performance metrics-average throughput, self-fairness, and Jain’s fairness index-are evaluated using the parameters summarized in Table 2.

**Figure 3:**
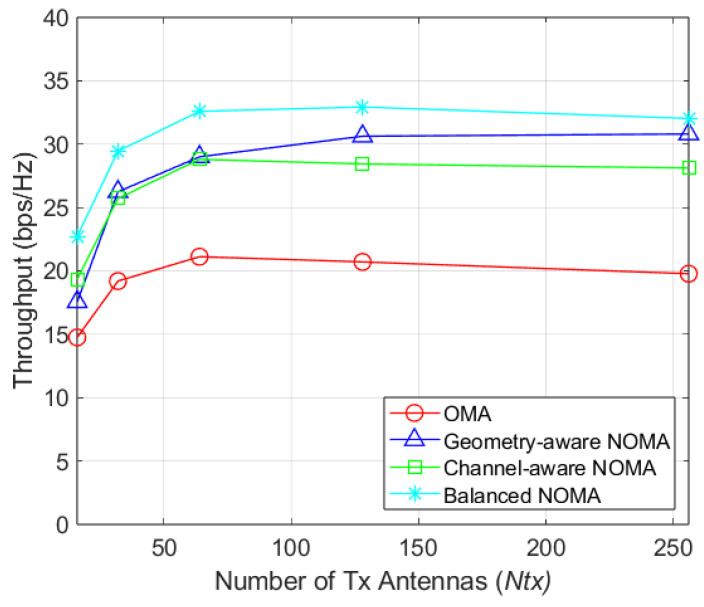
Throughput by number of transmit antennas (K=128).

Figure 3 illustrates the average throughput versus the number of transmit antennas ($N_{T}$) for a single-cell system with 8 RF chains and 128 users. While narrowing the beamwidth increases the channel gain, it also reduces the probability of two users being assigned to the same beam, creating a trade-off. In the proposed NOMA-OHBF system, the maximum average throughput is achieved at $N_{T}=64$ across all clustering methods. At this point, the proposed scheme achieves 43.39% higher throughput compared to $N_{T}=16$ case. Furthermore, it outperforms OMA by approximately 54.29%, nearly doubling the throughput and indicating that superimposed signals are effectively separated by SIC. While channel-aware clustering yields lower throughput than geometry-aware clustering, the proposed clustering method demonstrates a 12.37% improvement over the geometry-aware approach at $N_{T}=64$ (and 3.99% at $N_{T}=256$). This highlights the advatage of utilizing SINR feedback, which is more practical and effective than relying on explicit channel gain or AoD feedback.

**Figure 4:**
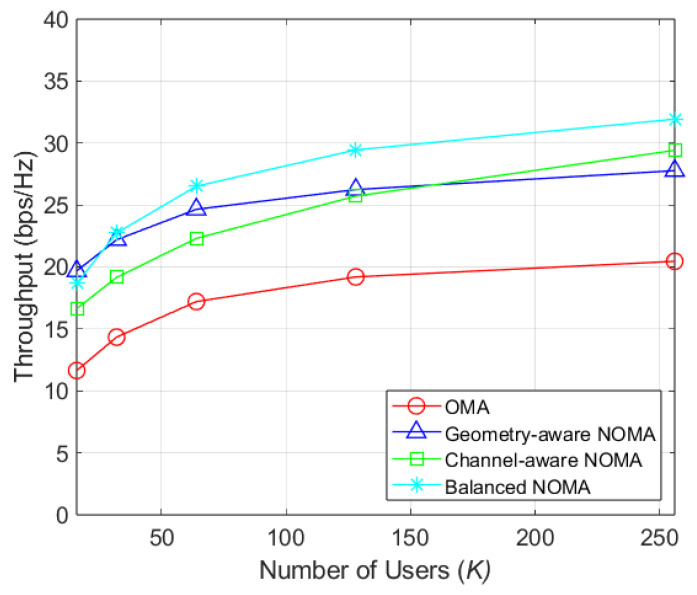
Throughput by number of users ($N_{T}=64$).

Figure 4 shows the average throughput as a function of the number of users (*K*) for $N_{T}=64$. In OBF systems, performance improves as more users enter the beam coverage. Consequently, as *K* increases, the probability of capturing users within a beam rises, leading to higher throughput. While channel-aware clustering requires a large user population to surpass geometry-aware clustering, the proposed scheme maintain stable performance even with small population (16 and 32 users) and exceeds geometry-aware clustering by more than 7.57% as the number of users grows.

**Figure 5:**
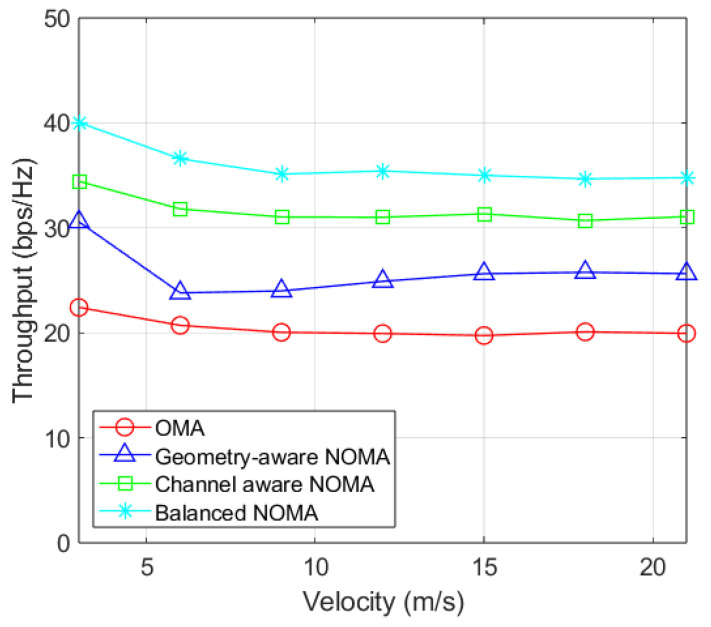
Throughput by user velocity ($N_{T}=64$ and K=256).

Figure 5 evaluates the system throughput against user velocity to validate the adaptivity of the memory-based approach. At a walking speed of 3 m/s, the proposed balanced-NOMA achieves a throughput of 40.0375 bps/Hz, significantly outperforming channel-aware NOMA (34.4347 bps/Hz), geometry-aware NOMA (30.5721 bps/Hz), and OMA (22.4207 bps/Hz). As velocity increases to 21 m/s, the throughput of the proposed scheme decreases by approximately 13.12% due to the doppler effect. Nevertheless, it maintain usperior robustness, remaining 11.95%, 35.68%, and 74.36% higher than the respectiv reference schemes. These results confirm that the adaptive perturbation and memory-based beam tracking effectively mitigate temporal channel variations in high-mobility scenarios.

**Figure 6:**
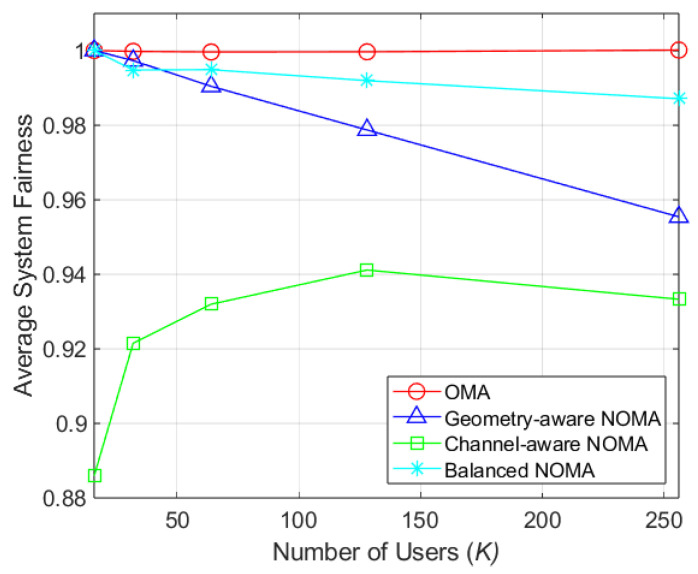
Self-fairness by number of users ($N_{T}=64$).

Figure 6 illustrates the self-fairness, representing the equality of user selection within a cell, as a function of the number of users (*K*) for $N_{T}=64$. In OBF-based systems, a PF-metric is applied to penalize previously served users, preventing their repeated selection in subsequent clustering or scheduling. In channel-aware clustering, which simultaneously pairs high-SINR and low-SINR users, the PF-metric often fails to operate effectively; the penalty assigned to a high-SINR user may inadvertently favor their selection as a low-SINR candidate, leading to redundant scheduling. In contrast, the proposed clustering method ensures the proper functioning of the PF-metric, achieving 1.35% higher self-fairness than geometry-aware clustering at K=128. This demonstrates that the proposed clustering method can maintain fairness in user selection while still prioritizing users with high SINR.

**Figure 7:**
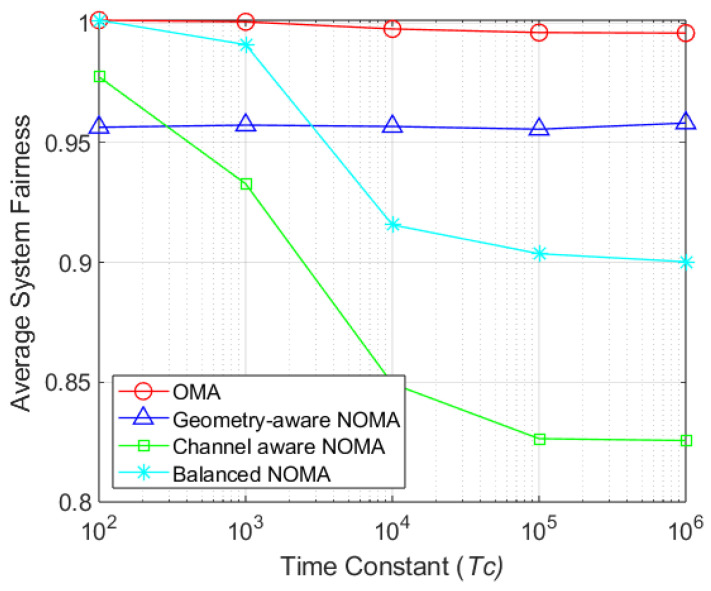
Self-fairness by time constant ($N_{T}=64$ and K=256).

Figure 7 illustrates the average system fairness as a function of the time constant $T_{c}$. The fairness of the proposed balanced-NOMA decreases by approximately 9.14% as $T_{c}$ increases from $10^{3}$ to $10^{6}$. Although its fairness at $T_{c}=10^{6}$ is about 6.04% lower than that of the geometry-aware NOMA, it consistently maintains a high fairness index above 0.9. Notably, under the same feedback overhead constraints where only SINR information is available, the proposed scheme achieves a 9.05% higher fairness index than channel-aware NOMA, which falls to 0.8256. These results confirm that the proposed scheme effectively mitigates the fairness degradation inherent in opportunistic strategies, providing robust and equitable resource allocation across various $T_{c}$ values.

**Figure 8:**
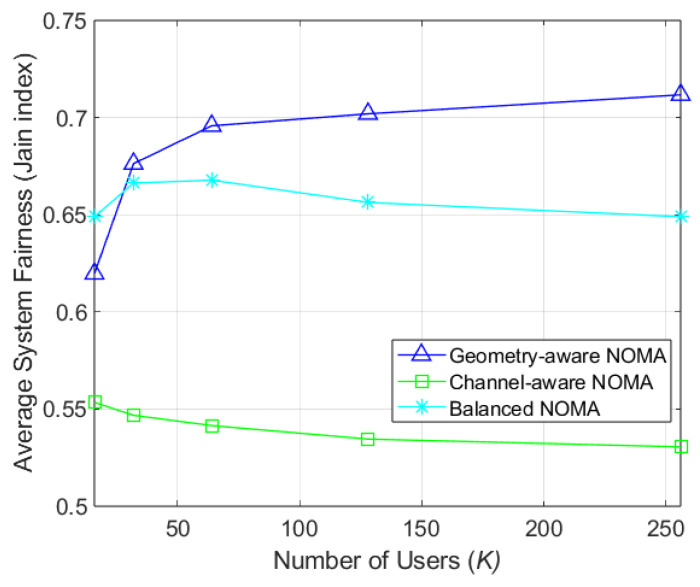
Jain’s index by number of users ($N_{T}=64$).

Figure 8 depicts Jain’s fairness index, which quantifies the throughput disparty between users within a single cluster. In NOMA, significant throughput differences are often necessary to facilitate SIC. At K=128, the proposed clustering method achieves a Jain’s index 22.78% higher than channel-aware clustering, though it remains 6.95% lower than geometry-aware approach. However, for K=16, the proposed method outperforms the geometry-aware scheme by 4.71%. Remarkably, the proposed clustering exhibits a maximum Jain’s index deviation of only 0.017 across different user populations, whereas the geometry-aware scheme shows a deviation of 0.092, indicating instability in dynamic environments. These finding demonstrate that the proposed clustering method maintains stable intra-cluster fairness while ensuring the performance required for robust SIC.

**Figure 9:**
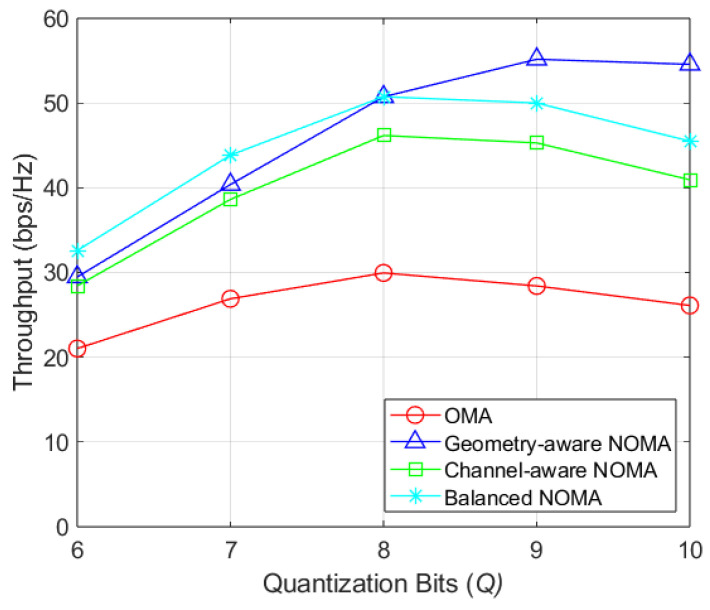
Throughput by quantization bits of codebook.

Figure 9 shows the average throughput versus the number of quantization bits in the codebook-based analog beamforming. As the quantization bits increases, beams become narrower, typically requiring more transmit antennas. For 8 bits or fewer, the proposed method achieves higher throughput than the reference schemes, indicating its effectiveness even in systems with limited antenna resources.

## 5. Conclusions

In this paper, we have proposed a balanced user clustering and PA framework for downlink mmWave MU-MIMO-NOMA systems operating under partial CSI. Unlike conventional NOMA schemes that rely on explicit CSI, the proposed approach utilizes an OBF framework based solely on SINR feedback, significantly reducing feedback overhead. Specifically, by integrating a memory-based greedy clustering algorithm with a SIC-aware PA strategy, we successfully addressed the inherent trade-off between system throughput and user fairness.

Numerical results confirm that the proposed scheme consistently outperforms conventional geometry-aware and channel-aware clustering strategies. Notably, the proposed method achieved up to a 12.37% improvement in average throughput and demonstrated superior robustness in high-mobility scenarios by maintaining stable intra-cluster and self-fairness indices. Furthermore, the effectiveness of the proposed scheme even under limited antenna resources and quantization bits highlights its practical feasibility for real-world deployments. Ultimately, this study provides a scalable and efficient solution for enhancing the performance of NOMA in next-generation mmWave networks where perfect CSI is unattainable.

## Figures and Tables

**Table 2:** System Parameters and Simulation Settings for NOMA Performance Evaluation.

Symbol	Description	Value
$N_{RF}$	Number of RF chain	8
$N_{T}$	Number of Antennas at BS	64 (Variable)
$N_{R}$	Number of Antennas at user	4
*K*	Number of Users	128 (Variable)
*d*	Antenna Spacing	λ/2
*Q*	Quantization Bits of RF precoder	6 (Variable)
*T*	Duration of Time Slot	1 ms
$N_{0}$	Signal to Noise Ratio	0 dB
*M*	Memory Size	20
$f_{c}$	Carrier Frequency	28 GHz
*L*	Number of Propagation Paths	4
$v_{UE}$	Moving Speed of Mobile Users	3 km/h
$\sigma_{\phi }^{2}$	Angle Variation	${\left(\pi /360\right)}^{2}$

## Data Availability

All data generated or analyzed during this study are included in this published article.

## Appendix A

The notations of this paper are shown in Table A1.

**Table A1:** System Parameters and Simulation Settings for NOMA Performance Evaluation.

Notation	Description	Notation	Description
$N_{RF}$	number of RF chain	$\alpha_{k,l}\left[t\right]$	complex gain of k-th user and l-th ray at t-th time slot
$N_{T}$	number of antennas at BS	$\phi_{k,l}\left[t\right]$	AoA of k-th user and l-th ray at t-th time slot
$N_{R}$	number of antennas at user	$\varphi_{k,l}\left[t\right]$	AoD of k-th user and l-th ray at t-th time slot
*K*	number of users	$F_{RF}$	radio frequency precoder matirx
*C*	number of clusters	$F_{BB}$	baseband precoder matirx
$N_{s}$	number of data streams	$PF_{k,s}^{\left(m\right)}$	PF-metric of k-th user on s-th data stream stored in m-th memory
$K_{n}$	number of users in n-th cluster	${\bar{R}}_{k}$	average throughput of the *k*-th user
$SINR_{n,k}$	signal to interference and noise of k-th user in n-th cluster	Γ	sum of PF metrics
$P_{n,k}$	allocated power of k-th user in n-th cluster	U	set of users
$w_{n,k}$	combiner vector of k-th user in n-th cluster	S	set of data streams
$H_{n,k}$	channel matrix of k-th user in n-th cluster	C	set of clusters
$f_{n}$	beforming precoder vector of n-th cluster	$T_{c}$	time constant
$R_{n,k}$	throughput of k-th user in n-th cluster	$g_{n,k}$	effective channel gain of k-th user in n-th cluster
*B*	transmission bandwidth	*M*	memory size
$F_{self}$	self-fairness	$p_{tol}$	minimum power for decoding SIC
$p_{k}$	probability that the k-th user is selected for transmission	$f_{D}$	doppler frequency
*J*	jain’s fairness index	ρ	time correlation coefficient
*L*	number of pathloss	$\sigma_{\phi }^{2}$	angle variation
